# Targeting ferroptosis in sensorineural hearing loss: mechanisms, therapeutics, and translational prospects

**DOI:** 10.3389/fneur.2026.1763297

**Published:** 2026-05-01

**Authors:** Lihong Zhang, Peiyu Luo, Xinghong Liu, Tao Guo, Hui Xie

**Affiliations:** 1 Department of Otorhinolaryngology, Chengdu University of Traditional Chinese Medicine, Chengdu, China; 2 Department of Otorhinolaryngology, Hospital of Chengdu University of Traditional Chinese Medicine, Chengdu, China

**Keywords:** ferroptosis, GPx4, hearing loss, lipid peroxidation, otoprotection

## Abstract

Sensorineural hearing loss (SNHL) is a major cause of disability worldwide, characterized by irreversible damage to cochlear hair cells and spiral ganglion neurons. Current treatments such as hearing aids and cochlear implants do not restore biological function. Ferroptosis, an iron-dependent form of regulated cell death driven by lipid peroxidation, has emerged as a key mechanism in acquired SNHL, including drug-, noise-, and age-related forms. This review systematically outlines the core molecular pathways of ferroptosis in SNHL, summarizes recent advances in ferroptosis-targeted interventions, and critically discusses current challenges and translational prospects. Although still largely preclinical, targeting ferroptosis represents a promising strategy for developing otoprotective therapies. Future research integrating novel technologies such as nano-delivery systems and single-cell omics may accelerate clinical translation.

## 1 Introduction

Sensorineural hearing loss (SNHL) is one of the most prevalent disabling sensory disorders worldwide (1) and represents the most common type of hearing impairment. SNHL typically refers to hearing loss resulting from lesions in the inner ear, involving structures such as cochlear hair cells, stria vascularis, spiral ganglion neurons, and the auditory nerve. When the sensory structures of the cochlea are damaged (2), mechanical vibrations transmitted from the middle ear cannot be converted into bioelectrical signals, i.e., neural impulses, leading to a failure of sound perception by the brain and consequently causing hearing loss. This condition severely impairs daily communication (3) and has profound consequences for mental health, social relationships, and overall quality of life.

The etiology of SNHL primarily includes natural aging (4), excessive noise exposure (5), Meniere’s disease (6), acoustic neuroma (7), among others. For relatively mild hearing loss, the cochlea’s inherent self-repair capacity (8) can enable some patients to recover their pre-loss hearing thresholds without pharmacological intervention. However, the regenerative ability of cochlear hair cells is limited; in cases of severe hearing loss, the chances for complete hearing recovery are slim. Although the inner ear was historically considered an immune-privileged organ, inflammation and oxidative stress are now recognized as key pathogenic mechanisms in hearing loss (9, 10). Irreversible damage or loss of inner ear hair cells (HCs) and spiral ganglion neurons (SGNs) is regarded as the primary pathological basis of SNHL (11). Currently, mainstream interventions for SNHL mainly involve fitting patients with suitable hearing aids or cochlear implants. While both modalities show significant efficacy in improving hearing-related quality of life (12, 13), hearing aids essentially rely on the impaired auditory system and are less effective for severe-to-profound hearing loss. Cochlear implants, on the other hand, are mechanical devices that directly electrically stimulate the auditory nerve. Both approaches have their respective limitations; they serve as functional substitutes rather than fundamentally repairing or regenerating damaged auditory cells. Therefore, it is necessary to investigate the mechanisms of SNHL and identify protective targets for HCs and SGNs to develop therapeutic strategies.

Currently, extensive research on SNHL has focused on programmed cell death (PCD) pathways, such as ferroptosis, necroptosis, and pyroptosis. Apoptosis, the first identified form of PCD (14) and the most extensively studied PCD pathway, contributes to the death of HCs and SGNs in SNHL (15, 16). Ferroptosis is an iron-dependent, novel form of regulated cell death (RCD) distinct from apoptosis, necrosis, and autophagy. Its primary biochemical features are the excessive accumulation of lipid peroxides (PL-OOHs) and the imbalance of intracellular antioxidant systems, such as GPX4. Currently, ferroptosis has been widely studied in fields including neurodegenerative diseases, ischemia–reperfusion injury, and tumors (17, 18). However, the link between ferroptosis and SNHL remains relatively unexplored.

This review aims to: systematically delineate the core mechanistic roles of ferroptosis in the pathogenesis and progression of various forms of acquired SNHL (such as drug-induced, noise-induced, and age-related hearing loss); comprehensively summarize the advances in drugs and therapeutic strategies targeting ferroptosis from preclinical studies; critically analyze the current controversies and challenges in this field; and prospectively discuss its future translational potential for clinical applications.

## 2 The core molecular mechanism of ferroptosis

**Figure 1:**
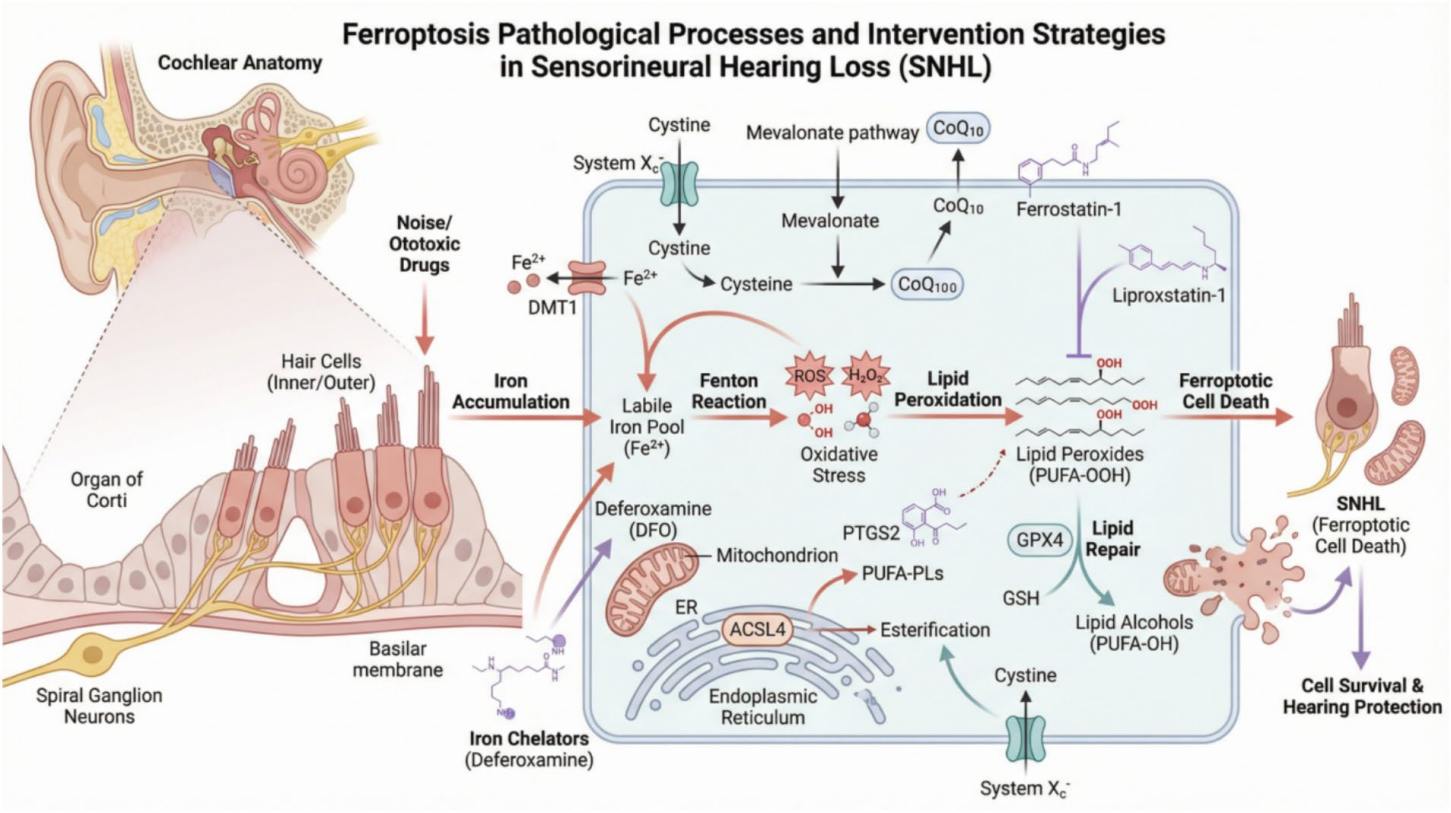
The key molecular pathways of ferroptosis (drawn by PL, XL).

Ferroptosis was first proposed between 2012 and 2013 as a novel, iron-dependent form of PCD triggered by excessive lipid peroxidation (17). Its unique biochemical essence lies in the peroxidative collapse of polyunsaturated fatty acid-containing phospholipids (PUFA-PLs) in the cell membrane, a process tightly regulated by three major systems: lipid metabolism, iron homeostasis, and the antioxidant defense network. Ferroptosis does not occur instantaneously; rather, it is a cascading process comprising initial stress, signal amplification, and final execution. Imbalance in iron metabolism, through mechanisms such as the Fenton reaction, iron overload, or impaired iron utilization, induces excessive generation of reactive oxygen species (ROS) (19), leading to oxidative stress damage. ROS disrupt lipid metabolic balance, resulting in oxidative stress and the production of membrane lipid peroxides (20). Concurrently, an imbalance in the amino acid antioxidant system hinders the clearance of excess lipid peroxides (21). These interconnected steps interact and collectively drive the initiation and progression of ferroptosis (Figure 1).

The core of lipid peroxidation is the peroxidation chain reaction of polyunsaturated fatty acid-containing phospholipids (22). Intracellular free polyunsaturated fatty acids, such as arachidonic acid (AA) and adrenic acid (AdA), must first be activated into acyl-CoA derivatives under the catalysis of acyl-CoA synthetase long-chain family member 4 (ACSL4) (23). Subsequently, lysophosphatidylcholine acyltransferase 3 (LPCAT3) integrates these activated polyunsaturated fatty acids into membrane phospholipids, providing the “substrate” for peroxidation (23, 24). Therefore, the activities of ACSL4 and LPCAT3 collectively determine a cell’s sensitivity to ferroptosis. The expression level of ACSL4 has been widely used as a key biomarker for cellular ferroptosis sensitivity. The polyunsaturated fatty acids integrated into membrane phospholipids undergo peroxidation catalyzed by various oxidases. Members of the lipoxygenase (ALOXs) family, particularly ALOX15 (25), and cytochrome P450 oxidoreductase (POR) (26), play crucial roles in this process by directly catalyzing the peroxidation of PUFAs, generating lethal lipid peroxides.

Iron, serving as an efficient catalyst for the Fenton reaction, is the origin of the term “ferroptosis” and the key driver for the explosive amplification of lipid peroxidation (17). Extracellular ferric iron (Fe^3+^) binds to transferrin and enters the cell via transferrin receptor 1 (TFR1)-mediated endocytosis. Within endosomes, Fe^3+^ is reduced to ferrous iron (Fe^2+^) and released into the cytosol through the divalent metal transporter 1 (DMT1), forming the labile iron pool. Excess iron is stored in an inert form within ferritin (18). Under ferroptosis-inducing signals, the ferritinophagy pathway mediated by nuclear receptor coactivator 4 (NCOA4) is activated. NCOA4, acting as a selective autophagy receptor, directs ferritin to autolysosomes for degradation, thereby rapidly releasing a large amount of free iron. This drastically expands the labile iron pool, catalyzes the Fenton reaction, explosively generates a massive amount of reactive oxygen species, and ultimately leads to lipid peroxidation (18, 27).

Cells possess multiple layers of anti-ferroptosis defense systems, whose common function is to scavenge lipid peroxides and maintain the redox homeostasis of the cell membrane. The failure of these systems is a prerequisite for the occurrence of ferroptosis. Glutathione peroxidase 4 (GPX4) is the most important ferroptosis inhibitor identified to date (18). It directly utilizes glutathione to reduce lipid peroxides into non-toxic lipid alcohols (28), thereby blocking the propagation of lipid peroxidation at its source.

The ferroptosis suppressor protein 1/coenzyme Q10 (FSP1/CoQ10) system is a key anti-ferroptosis backup defense system, independent of the GPX4/GSH axis, discovered in recent years. Guided by its plasma membrane localization signal, FSP1 (Ferroptosis Suppressor Protein 1) localizes to the plasma membrane and utilizes NAD(P)H to reduce the oxidized form of ubiquinone (coenzyme Q10, CoQ10) to its hydroquinone form (CoQ10H₂). As a potent lipophilic antioxidant, CoQ10H₂ can directly capture lipid radicals, thereby interrupting the propagation chain of lipid peroxidation within the cell membrane (29). This system is particularly important under conditions of GPX4 inactivation or GSH depletion, providing cells with an additional crucial layer of antioxidant protection (21). Studies have shown that the expression level of FSP1 is closely associated with cellular sensitivity to ferroptosis, and its overexpression can significantly confer resistance to ferroptosis induced by GPX4 inactivation (21, 30). Therefore, the GPX4/GSH and FSP1/CoQ10 systems collectively constitute an endogenous “dual-safeguard” mechanism for cellular defense against ferroptosis.

Given this understanding of the intricate mechanisms of ferroptosis, we must further investigate its connection with SNHL. Currently, mounting evidence indicates a close association between the two, with oxidative stress and lipid peroxidation playing crucial roles in the onset and progression of SNHL. Therefore, investigating ferroptosis offers a novel perspective and potential therapeutic targets for the prevention and treatment of SNHL, holding significant theoretical value and clinical translation potential.

## 3 Evidence for and role of ferroptosis in SNHL

**Figure 2:**
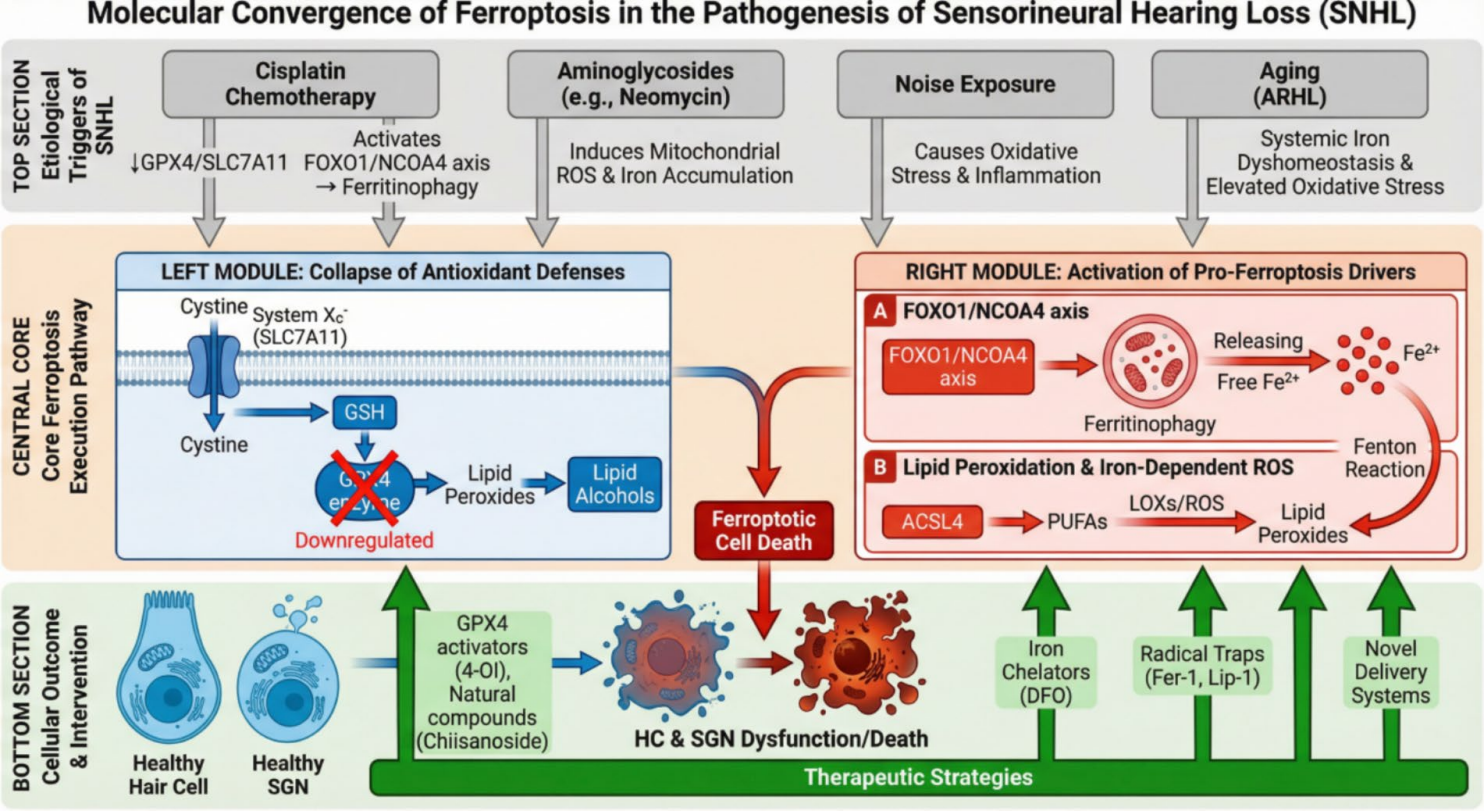
Key molecular pathways involved in ferroptosis and sensorineural hearing loss. This figure summarizes the key molecular pathways implicated in hearing loss induced by cisplatin, aminoglycosides, aging, and noise exposure (drawn by TG, XL).

Since the concept of ferroptosis was proposed, a growing body of research has increasingly focused on exploring its correlation with SNHL. As an iron-dependent, lipid peroxidation-driven novel form of cell death, ferroptosis has been demonstrated to play a pivotal role in the pathogenesis of various acquired forms of SNHL, such as drug-induced, noise-induced, and age-related hearing loss, as highlighted in recent studies (21). These investigations have not only revealed the close association between ferroptosis and SNHL but have also laid the groundwork for a deeper understanding of its underlying mechanisms. Figure 2 summarizes the key molecular pathways associated with ferroptosis and hearing loss identified in these studies.

### 3.1 Drug-induced hearing loss

Drug-induced hearing loss, particularly that triggered by agents such as cisplatin and neomycin, serves as the most paradigmatic model for studying the role of ferroptosis in SNHL (31). Recent studies have indicated an association between ferroptosis and ototoxicity caused by cisplatin, neomycin (32), hydroperoxides, and free fatty acids (FFAs) (11). Among these, the role of ferroptosis in cisplatin-induced ototoxicity has been the most extensively studied (33).

#### 3.1.1 The anticancer drug cisplatin

Cisplatin is a widely used chemotherapeutic anticancer drug for treating solid malignant tumors in both children and adults, currently indicated for various malignancies including lung cancer, testicular cancer, ovarian cancer, and head and neck cancers (34). However, its clinical benefit is often limited by ototoxicity, leading to irreversible sensorineural hearing loss (35, 36). Statistics show that a considerable proportion of patients receiving cisplatin therapy develop irreversible sensorineural hearing loss. This hearing impairment is particularly pronounced in the high-frequency region and worsens with cumulative chemotherapy cycles (36, 37). Cisplatin’s ototoxicity primarily manifests as specific damage to HCs and SGNs (38). As the primary neurons for auditory signal transmission, the survival status of SGNs directly affects the function of the auditory conduction pathway and even determines the efficacy of cochlear implantation. The traditional view holds that cisplatin primarily induces auditory cell damage through oxidative stress and apoptosis (39). However, in recent years, accumulating evidence suggests that ferroptosis, a newly discovered form of cell death, plays a crucial role in cisplatin-induced ototoxicity.

Morphologically distinct from other forms of cell death such as apoptosis and necrosis, ferroptosis is characterized by a reduction or disappearance of mitochondrial cristae and increased membrane density, while maintaining normal nuclear size. Cisplatin has been found to directly or indirectly interfere with GPX4 function in auditory cells, thereby initiating the ferroptosis program (11).

Cisplatin disrupts the cellular antioxidant defense system primarily by downregulating the SLC7A11-GPX4 axis (31, 40). Both mRNA and protein levels of SLC7A11 and GPX4 are significantly reduced in cochlear hair cells and spiral ganglion neurons following cisplatin treatment (41). This suppression limits cysteine uptake and glutathione synthesis, leading to GPX4 inactivation and subsequent accumulation of lipid peroxides—a central execution step of ferroptosis as detailed in Section 2 (42).

In cisplatin-induced auditory neuron damage, Wang et al. (43) identified a crucial regulatory role of the FOXO1-NCOA4 axis. Their research demonstrated that cisplatin treatment significantly upregulates the expression of NCOA4 in spiral ganglion neurons and enhances the interaction between NCOA4 and ferritin, leading to ferritin degradation and the release of free iron. Notably, they further revealed that the transcription factor FOXO1 acts as an upstream regulator of NCOA4. Under normal conditions, FOXO1 binds to the promoter region of the NCOA4 gene, suppressing its transcriptional expression. However, cisplatin treatment impedes the nuclear translocation of FOXO1, preventing it from effectively repressing NCOA4 transcription. This releases the inhibition on NCOA4, resulting in its upregulated expression (43).

Cisplatin orchestrates auditory cell ferroptosis via two synergistic molecular pathways: on one hand, by downregulating SLC7A11 and GPX4 to disrupt the cellular antioxidant defense capacity; on the other hand, by activating the FOXO1-NCOA4 axis to trigger ferritinophagy, leading to iron overload (31, 41, 43). These two pathways do not operate independently but rather reinforce each other, forming a vicious cycle. The accumulation of lipid peroxides resulting from GPX4 inactivation may further promote NCOA4-mediated ferritinophagy, while the free iron released by ferritinophagy, in turn, exacerbates lipid peroxidation, ultimately leading to irreversible damage in auditory cells.

Beyond directly inhibiting ferroptosis, enhancing the cellular antioxidant defense capacity also represents an effective strategy. Studies have found that the natural compound madecassic acid (MA) can inhibit ferroptosis by upregulating GPX4 expression, thereby protecting vestibular hair cells and improving balance function (44).

Cisplatin induces ferroptosis in HCs and SGNs by coordinately targeting the SLC7A11-GPX4 and FOXO1-NCOA4 axes, which constitutes a crucial mechanism of its ototoxicity (31, 41, 43). On one hand, cisplatin downregulates SLC7A11 and GPX4, thereby disrupting the cellular antioxidant defense and leading to the accumulation of lipid peroxides. On the other hand, it activates ferritinophagy by relieving FOXO1-mediated transcriptional repression of NCOA4, resulting in iron overload. These two pathways act synergistically, converging to drive auditory cells toward ferroptosis. A deeper understanding of this molecular network provides a theoretical foundation and potential targets for developing novel protective strategies against cisplatin-induced ototoxicity. Future research should focus on translational medicine, exploring feasible approaches to translate these molecular targets into clinical applications, aiming to mitigate chemotherapy-associated hearing loss and improve the quality of life for cancer patients.

#### 3.1.2 Neomycin

Neomycin, a classic aminoglycoside antibiotic, has its significant ototoxic side effects recognized as a major cause of drug-induced hearing loss. Recent studies have revealed that ferroptosis—the iron-dependent, lipid peroxidation-driven novel form of cell death—plays a central role in neomycin-induced damage to auditory hair cells.

Neomycin exposure induces oxidative stress in the cochlea, leading to mitochondrial-derived lipid ROS accumulation and subsequent peroxidation of membrane polyunsaturated fatty acids (32). This process is amplified by concurrent accumulation of ferrous ions, which catalyze Fenton reaction-driven lipid peroxidation, thereby propagating ferroptotic cell death (45).

Notably, a 2024 study published in Cell and Bioscience (46), utilizing a zebrafish lateral line hair cell model, was the first to confirm through chemical screening that inhibiting the intracellular ferroptosis pathway is an effective strategy for alleviating neomycin-induced ototoxicity. This study screened seven small-molecule compounds from a natural alkaloid library, all of which protected hair cells from neomycin damage by intervening in the ferroptosis pathway. Among them, six acted as radical-trapping agents, while the other one (ellipticine) functioned by modulating intracellular iron homeostasis.

Regarding interventional measures, the ferroptosis-specific inhibitor Liproxstatin-1 (Lip-1) has been confirmed to effectively mitigate neomycin-induced ototoxicity (47). Its protective mechanism may involve scavenging lipid peroxides, blocking the propagation of lipid peroxidation, and maintaining intracellular redox homeostasis. Furthermore, studies have found that Lip-1 also exerts its protective effect by inactivating lysosomal iron, suggesting that the stability of the lysosomal iron pool may play a critical regulatory role in hair cell ferroptosis.

These findings not only deepen our understanding of the mechanisms underlying neomycin-induced ototoxicity but also provide novel potential targets for the clinical prevention and treatment of drug-induced hearing loss. Targeting the ferroptosis pathway, particularly through strategies such as utilizing ferroptosis inhibitors like Lip-1, holds promise as an effective future strategy for protecting auditory function and mitigating the ototoxicity associated with aminoglycoside antibiotics.

### 3.2 Noise-induced hearing loss

Noise-induced hearing loss (NIHL) constitutes one of the major causes of SNHL, and its occurrence is closely associated with irreversible damage to cochlear hair cells (HCs) and spiral ganglion neurons (SGNs). In recent years, ferroptosis, a newly identified form of regulated cell death, has been confirmed to play a critical pathogenic role in NIHL. In today’s highly urbanized and fast-paced societal life, the impact of noise on hearing is difficult to avoid and cannot be overlooked.

Intense noise exposure, the primary initiating factor for NIHL, induces immediate cochlear vasoconstriction and ischemia, thereby causing transient hypoxia. Subsequent reperfusion triggers excessive ROS production, leading to oxidative stress and hair cell damage (48). Noise induces oxidative stress and inflammatory responses within the cochlea through dual pathways—acoustic trauma and metabolic stress—which in turn drive the occurrence of ferroptosis. High-intensity noise can lead to massive generation of ROS in the cochlea, initiating a chain reaction of lipid peroxidation (21). Simultaneously, noise exposure activates inflammatory pathways such as nuclear factor kappa B (NF-κB) (49, 50), promoting the release of pro-inflammatory factors including tumor necrosis factor-alpha (TNF-α) and interleukin-1β (IL-1β), which further exacerbates oxidative damage. These processes collectively result in disrupted iron metabolism and the collapse of the antioxidant defense system within the cochlea, manifested as iron ion accumulation, glutathione depletion, and inhibition of glutathione peroxidase 4 (GPX4) activity (21).

Noise exposure upregulates lipid peroxidation markers such as malondialdehyde (MDA) and 4-hydroxynonenal (4-HNE) in cochlear tissues, compromising hair cell membrane integrity (21). Ferrostatin-1 (Fer-1), a radical-trapping antioxidant, has shown significant protective effects in NIHL models by neutralizing lipid peroxyl radicals and interrupting the propagation of lipid peroxidation (51). Furthermore, the secondary amine nitrogen atom within the Fer-1 molecule can bind to ferrous ions; after being oxidized, it can be reduced back by intracellular ferrous ions, forming a “pseudo-catalytic cycle.” This allows a minimal amount of Fer-1 to sustain its antioxidant activity continuously. Additionally, the lipophilic nature of Fer-1 enables it to embed into membrane regions rich in polyunsaturated fatty acids, particularly within the membrane structures of hair cells and spiral ganglion neurons, providing direct protection against oxidative damage.

Studies have shown that administration of Fer-1 either before or during the early stages after noise exposure can significantly alleviate noise-induced elevation of hearing thresholds and reduce hair cell loss (21). The role of ferroptosis in NIHL is not isolated; rather, it forms a complex network with other cell death pathways. Therefore, combining Fer-1 with other therapeutic strategies may yield synergistic protective effects. For instance, the combination of Fer-1 with antioxidants such as N-acetylcysteine can simultaneously target both ferroptosis and apoptotic pathways, providing more comprehensive protection. Alternatively, its combination with anti-inflammatory drugs like corticosteroids can concurrently suppress noise-induced inflammatory responses and ferroptosis, generating additive effects (52).

### 3.3 Age-related hearing loss

The systemic dysregulation of iron metabolism and the intensification of oxidative stress that occur with aging are considered key pathogenic factors in age-related hearing loss (ARHL), rendering the cochlea in elderly individuals more susceptible to ferroptosis.

During the aging process, the antioxidant defense capacity of the inner ear gradually diminishes. Meanwhile, increased generation of ROS leads to elevated levels of oxidative stress. Excess ROS attack polyunsaturated fatty acids in the cell membrane, initiating the chain reaction of lipid peroxidation, a hallmark event in the onset of ferroptosis. Moreover, aging is often accompanied by systemic dysregulation of iron homeostasis, which may result in abnormal iron accumulation in cochlear tissues (53). Excessive free ferrous ions can then catalyze the generation of more radicals from lipid peroxidation products via the Fenton reaction, further accelerating the ferroptosis process in cochlear hair cells and spiral ganglion neurons (54).

Based on the aforementioned mechanisms, researchers have explored pharmacological intervention strategies to delay ARHL. Pham et al. (55) demonstrated that the compound CMS121 exhibits protective effects in ARHL models, such as the SAMP8 senescence-accelerated mouse. It significantly reduces auditory brainstem response (ABR) threshold shifts and enhances protection for inner hair cell ribbon synapses by mitigating inflammatory responses and excessive lipid peroxidation within the cochlea. This suggests that inhibiting ferroptosis-related pathways may effectively slow the progression of ARHL. Okur et al. (56) showed that long-term supplementation with nicotinamide riboside (NR), a precursor of NAD^+^, prevents the progression of ARHL in mice. Its mechanism is related to restoring cochlear NAD^+^ levels, enhancing synaptic transmission, and regulating the cochlear lipid droplet metabolism pathway. Research by Cho et al. (57) in cynomolgus monkeys found that long-term metformin administration delays cochlear aging, manifested as reduced hair cell loss and attenuated stria vascularis atrophy. In this study (based on the non-human primate aging model established by Yang et al.), metformin was administered orally at 20 mg/kg daily for 40 months (approximately 3.3 years), a regimen equivalent to the standard clinical dose for diabetes treatment in humans and corresponding to approximately 10 years of human aging (57, 58). The molecular mechanisms involve downregulating inflammation-related genes while upregulating genes associated with sound perception and neural signaling.

### 3.4 Mechanistic comparison and integration of ferroptosis induced by various Etiologies

In summary, evidence for ferroptosis activation exists in drug-induced, noise-induced, and age-related sensorineural hearing loss (SNHL). However, a core scientific question remains: do these heterogeneous triggers ultimately converge on a common ferroptosis execution pathway? The comparative analysis in the table below (Table 1) explores this issue.

**Table 1:** Comparison of key mechanisms of ferroptosis in drug-, noise-, and age-related SNHL.

Comparison dimension	Drug-induced SNHL (e.g., cisplatin)	Noise-induced hearing loss (NIHL)	Age-related hearing loss (ARHL)
Primary insult & initial stress	Direct or indirect cellular action of ototoxic drugs.	Intense mechanical-metabolic stress (acoustic trauma) and ischemia–reperfusion.	Chronic, low-grade oxidative stress and systemic/local metabolic decline.
Primary source of iron overload	Ferritinophagy-driven: Significantly activated via the FOXO1/NCOA4 axis, releasing large amounts of free iron.	Secondary iron metabolism dysregulation: Possibly due to hemorrhage, inflammation, and heme degradation leading to iron release.	Progressive iron homeostasis imbalance: Systemic iron metabolism dysregulates with aging, leading to gradual cochlear iron accumulation.
Key trigger of lipid peroxidation	Direct collapse of antioxidant defense: Drugs directly or indirectly inhibit System Xc^−^ (SLC7A11) or GPX4, causing defense system “failure.”	Burgeoning production of reactive oxygen species (ROS): Noise causes dysfunction in mitochondria etc., generating excess ROS that directly attack lipids.	Progressive decline in antioxidant capacity: Activity of endogenous antioxidant enzymes (e.g., GPX4) decreases with age, impairing clearance capability.
Core pathway alterations	Dual hit of SLC7A11/GPX4 axis suppression and NCOA4-mediated ferritinophagy activation.	Driven jointly by intense oxidative stress and secondary inflammation, possibly involving upregulation of enzymes like LOXs.	Decline of multiple systems: Involving weakened antioxidant defense, reduced repair capacity, and persistent subclinical inflammation.
Primary cellular targets	Both hair cells (HCs) and spiral ganglion neurons (SGNs) are significantly affected.	Hair cells (particularly outer hair cells) are the primary targets, with secondary effects on SGNs.	All cochlear cell types are affected broadly by the aging process.
Mechanistic convergence points	All converge on the dysfunction of the GPX4/GSH antioxidant system and the lethal accumulation of lipid peroxides. Disordered iron metabolism (albeit via different routes) acts as a common amplifier.		
Unique or emphasized pathways	High dependence on ferritinophagy and direct inhibition of System Xc^−^.	Closely linked to acute energy crisis and physical damage, with a stronger inflammatory initiation signal.	Deeply associated with systemic and cellular senescence programs, representing a chronic, multifactorial cumulative process.

As summarized in Table 1, the three etiologies ultimately converge on a common endpoint: relative or absolute insufficiency of the GPX4/GSH system function within cochlear cells and the uncontrolled accumulation of lipid peroxides. This indicates that, despite differing upstream “triggers,” the core execution pathway of ferroptosis is shared. However, the primary routes leading to this endpoint vary. Agents like cisplatin appear more adept at proactively dismantling cellular antioxidant defenses (e.g., by inhibiting SLC7A11) and activating specific iron-release programs (ferritinophagy). In contrast, noise exposure resembles a saturation attack, overwhelming cellular clearance capacity with an outburst of ROS. Aging, meanwhile, is akin to a gradual erosion, accompanied by the synchronous decline of protective mechanisms at multiple levels. Therefore, strategies targeting the common endpoint (e.g., using broad-spectrum lipid peroxidation inhibitors) or key convergence nodes (e.g., stabilizing GPX4) may hold promise for broad-spectrum protection. Conversely, strategies targeting unique pathways (e.g., specifically inhibiting cisplatin-induced NCOA4 activation) could enable more precise, etiology-specific prevention and treatment.

### 3.5 Examining the role of ferroptosis in SNHL: strength of evidence, model limitations, and relative importance of mechanisms

Following the integrated comparison of mechanisms across different etiologies, a further methodological perspective on the strength of evidence, model limitations, and the relative importance of ferroptosis within the cell death network is crucial for evaluating the reliability and translational potential of these findings.

On one hand, the strength of evidence varies substantially across model systems. Mechanistic studies in cell lines (e.g., HEI-OC1) have established foundational links between ferroptosis regulators (GPX4, ACSL4, NCOA4) and ototoxic insults. However, these immortalized cells lack the native cochlear microenvironment and cannot recapitulate the complex cell–cell interactions or the blood-labyrinth barrier that modulate drug responses *in vivo*; rodent models have provided crucial *in vivo* validation through the use of specific inhibitors such as Ferrostatin-1 and Liproxstatin-1, which consistently attenuate hearing loss and hair cell damage across multiple injury paradigms (11, 47, 59). Yet, it must be noted that most rodent studies employ acute injury models (e.g., single high-dose cisplatin or intense noise exposure), which may not faithfully represent the chronic, progressive nature of human SNHL, particularly in age-related hearing loss. Direct clinical evidence establishing ferroptosis as a dominant mechanism in human SNHL remains scarce, with current data limited to retrospective biomarker analyses (e.g., serum iron markers, post-mortem tissue) that can only infer association, not causality (53, 60).

On the other hand, ferroptosis does not operate in isolation but intersects with other programmed cell death pathways in a context-dependent manner. In many SNHL models, ferroptosis does not occur in isolation. For instance, in cisplatin-induced ototoxicity, inhibiting apoptosis provides only partial protection, suggesting that other pathways (such as ferroptosis and necroptosis) may act in concert or sequentially (11). A 2025 study, by specifically manipulating this pathway using RSL3 (a ferroptosis inducer) and Ferrostatin-1 (a ferroptosis inhibitor), demonstrated that ferroptosis can directly cause hearing loss and may serve as the dominant mechanism under certain circumstances (11). Conversely, in noise-induced hearing loss, the intense inflammatory response may concurrently activate pyroptosis and ferroptosis, making it difficult to disentangle their relative contributions. In age-related hearing loss, ferroptosis likely operates as one component of a multifactorial degenerative process involving cumulative oxidative damage, mitochondrial dysfunction, and chronic inflammation, rather than as a singular triggering event. Thus, the primacy of ferroptosis varies by etiology and insult intensity, necessitating careful interpretation of inhibitor studies that may inadvertently capture cross-pathway effects.

The third aspect, the causal inference from current evidence is often limited by overlapping pathway crosstalk and compensatory mechanisms. Inhibitor-based approaches (e.g., Fer-1 for ferroptosis, necrostatin-1 for necroptosis) are widely used to infer pathway contributions, but these inhibitors may lack absolute specificity, and blocking one death pathway can shunt cells toward alternative routes. This compensatory plasticity complicates the interpretation of single-pathway interventions and underscores the need for genetic models with conditional, spatiotemporally controlled knockout of key ferroptosis regulators (e.g., GPX4, ACSL4) in specific cochlear cell types. Such approaches, combined with dynamic monitoring of multiple cell death markers, would provide a more definitive map of the ferroptosis landscape *in vivo*.

Finally, the translational gap between preclinical models and human disease remains substantial. Each model system carries inherent limitations that constrain extrapolation to human SNHL (Table 2). Cell lines lack systemic physiology; rodents differ in cochlear anatomy, lifespan, and metabolic regulation; non-human primate studies are prohibitively expensive and ethically complex; and human inner ear tissue is rarely accessible for mechanistic interrogation. This evidence hierarchy must be acknowledged when evaluating the strength of conclusions, and future research should prioritize the development of human-relevant models such as cochlear organoids or induced pluripotent stem cell-derived hair cells to bridge this gap (61).

**Table 2:** Comparison of different model systems in ferroptosis research for SNHL.

Model system	Advantages	Limitations	Implications and translational significance for ferroptosis research
Cell lines (e.g., HEI-OC1) (11, 51)	• Homogeneous cell population, facilitating mechanistic exploration • Suitable for high-throughput drug screening and genetic manipulation (103) • Low cost and short experimental cycles	• Lack *in vivo* microenvironment (e.g., blood-labyrinth barrier, fluid circulation) • Cannot assess overall auditory function (e.g., ABR thresholds) • Immortalized cells may deviate from primary cell physiology	• Conclusions require *in vivo* validation • Efficient platform for discovering core pathways (e.g., GPX4, SLC7A11) (103)
Rodent Models (Mice, Rats) (11, 51, 75)	• Fully simulate hearing threshold shifts and hair cell/neuron loss (47, 59) • Mainstream model for protective drug intervention studies (47, 59) • Genetically engineered mice enable specific gene function studies	• Differences in lifespan and metabolic rate vs. humans • Cochlear structure & auditory frequency range differ from humans • Difficult to replicate chronic progression of human SNHL (e.g., ARHL)	• Core *in vivo* validation platform for drug efficacy & safety (47, 59) • Consider species differences during clinical translation
Non-human primates (e.g., Cynomolgus Monkeys) (11)	• Cochlear anatomy, auditory physiology & aging highly similar to humans • Exceptionally high translational value for predicting human drug effects	• Extremely high cost & strict ethical review • Small sample sizes limit statistical power • Few existing studies challenge reproducibility	• Provide most compelling preclinical evidence • Suitable for studying complex, chronic processes like age-related hearing loss
Human tissues/clinical data (53, 58)	• Highest direct relevance, no species differences • Can identify diagnostic or prognostic biomarkers	• Living inner ear tissue hard to obtain (autopsy/rare surgery) (11) • Mostly retrospective, limiting causal establishment • Numerous confounding factors hard to control	• Ultimate bridge for validating animal model findings & linking mechanisms to disease • Drive translational & precision medicine development

### 3.6 Environmental ototoxicants: lead and per-and polyfluoroalkyl substances (PFAS)

Beyond the well-established etiologies of drug-, noise-, and age-related hearing loss, emerging evidence suggests that environmental ototoxicants may also contribute to SNHL. Among these, lead and per- and polyfluoroalkyl substances (PFAS) represent two classes of environmental contaminants with growing public health concern. However, unlike cisplatin, noise, and aging, the direct involvement of ferroptosis in lead- and PFAS-induced hearing loss remains largely unexplored.

Lead is a classic environmental neurotoxicant with well-documented ototoxic effects. A recent quantitative proteomics study revealed that chronic lead exposure induces cochlear oxidative stress and predominantly causes cochlear synaptopathy, characterized by ribbon synapse disruption and altered synaptic vesicle cycling, without affecting outer hair cell viability or causing hair cell loss (62). Building on this, direct evidence has now emerged demonstrating that lead exposure induces ferroptosis in spiral ganglion neurons, characterized by decreased GPX4 and SLC7A11 expression, increased iron accumulation and lipid peroxidation, and mitochondrial shrinkage; pre-treatment with ferroptosis inhibitors significantly attenuated lead-induced SGN injury (63). Given that oxidative stress is a key trigger of ferroptosis, it is plausible that ferroptosis may contribute to lead-induced hearing loss. However, whether these ferroptosis pathways operate in cochlear hair cells following lead exposure remains to be established.

Per- and polyfluoroalkyl substances (PFAS) are emerging persistent environmental contaminants with poorly understood ototoxic effects. Epidemiological studies have associated PFAS exposure with hearing impairment in US adults, particularly for perfluorononanoic acid (PFNA) and perfluorodecanoic acid (PFDA) (64, 65). A recent preprint study demonstrated that PFAS mixture exposure induces outer hair cell loss, ribbon synapse damage, and spiral ganglion neuron degeneration in mice (66). However, the molecular mechanisms underlying PFAS-induced cochlear damage—including whether ferroptosis is involved—remain largely unexplored and warrant further investigation.

**Figure 3:**
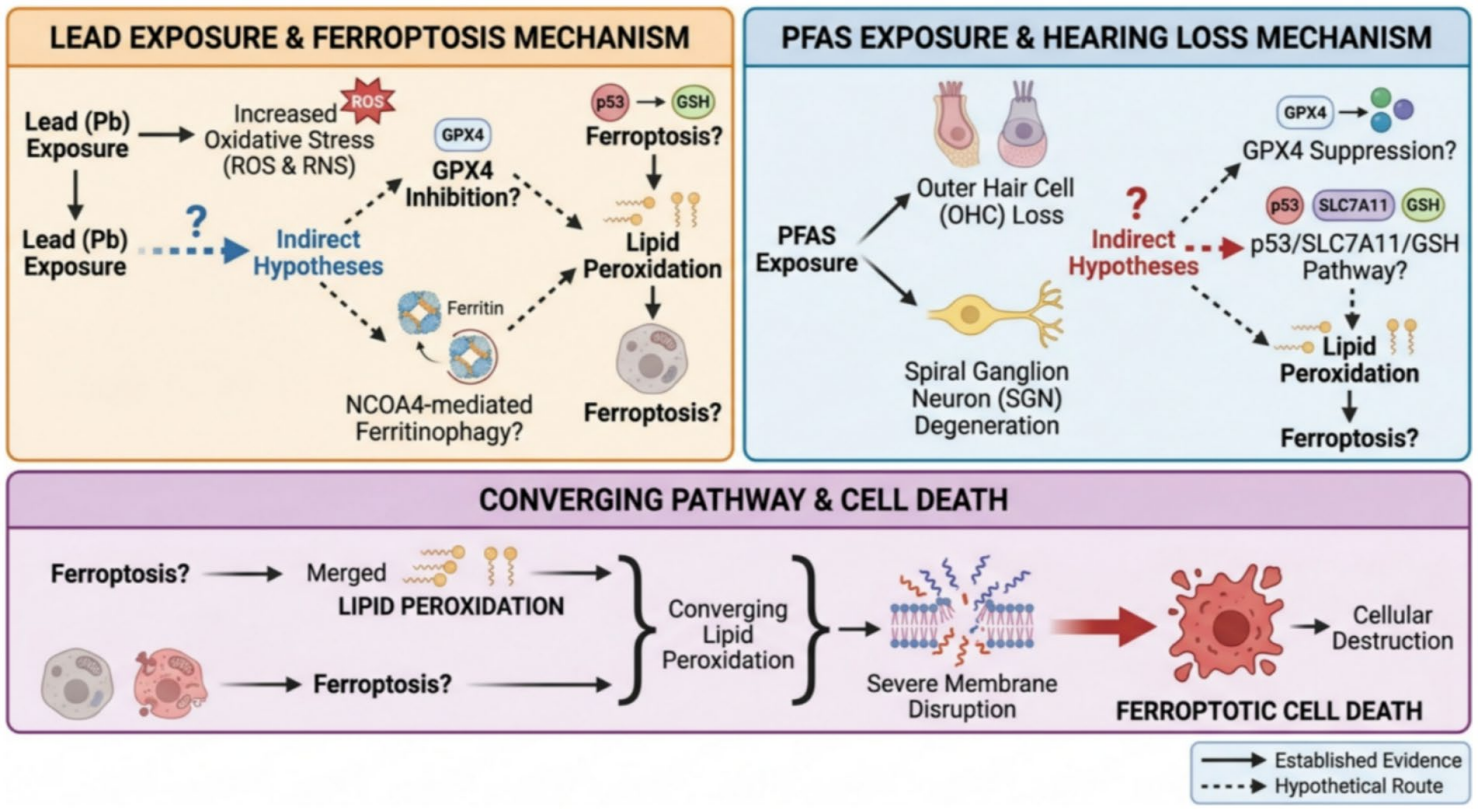
Proposed ferroptosis pathways in lead- and PFAS-induced hearing loss. Solid arrows = established [Bhatia et al. (62); Huang et al. (63); Ding and Park (64); Li (65); Bhatia et al. (66); dashed arrows = hypothetical; “?” = requires validation (drawn by TG, XL)].

A schematic diagram summarizing the hypothetical mechanisms by which lead and PFAS might induce ferroptosis is presented in Figure 3. Dashed arrows indicate proposed pathways requiring experimental validation.

## 4 Prevention and treatment strategies targeting ferroptosis

The causes of hearing loss are diverse, making the search for effective mitigation strategies a pressing issue. In this section, we review the current research progress on drugs or strategies that exert otoprotective effects by targeting ferroptosis, along with their underlying mechanisms.

### 4.1 Ferroptosis inhibitors

Based on the core molecular pathways of ferroptosis (as outlined in Section 2), multiple inhibitors exert otoprotective effects by intervening at different stages. According to their primary targets, they can be categorized into the following classes:

Iron Chelators, such as deferoxamine (DFO), function by specifically chelating excessive intracellular Fe^3+^, thereby reducing the pool of free iron available to catalyze lipid peroxidation. In cisplatin-induced ototoxicity models, DFO treatment significantly alleviates lipid peroxidation levels and cell death in hair cells. Additionally, DFO may enhance cellular adaptive stress responses by stabilizing hypoxia-inducible factor-1α (HIF-1α) (11, 67, 68).

Radical-Trapping Antioxidants, such as Ferrostatin-1 (Fer-1) and Liproxstatin-1 (Lip-1), function by directly neutralizing lipid peroxyl radicals, thereby effectively interrupting the chain reaction of lipid peroxidation. Both have demonstrated significant protective effects in various hearing loss models, such as noise-induced and drug-induced ototoxicity (21, 51, 69). Among them, Lip-1, as a next-generation inhibitor, exhibits enhanced metabolic stability and tissue penetrability.

GPX4 Activators/Stabilizers, such as 4-octyl itaconate (4-OI), enhance endogenous defense capacity by activating pathways like Nrf2, thereby upregulating the expression of antioxidant genes including SLC7A11 and GPX4. Studies have confirmed that 4-OI treatment significantly alleviates cisplatin-induced ferroptosis in cochlear hair cells (70).

Other Target Inhibitors: This category includes ACSL4 inhibitors (e.g., rosiglitazone), lipoxygenase (LOXs) inhibitors (e.g., zileuton), and NCOA4 inhibitors. They function by reducing the synthesis of peroxidation-susceptible lipids, blocking the initiation of lipid peroxidation, or inhibiting ferritinophagy, respectively (43, 71, 72).

The combined use of these different types of inhibitors may yield synergistic effects, offering a more effective strategy for hearing protection.

### 4.2 Natural compounds and drugs with anti-ferroptotic activity

In recent years, studies have found that various natural products, including lupane derivatives (such as Chiisanoside) and the flavonoid Luteolin, can effectively inhibit ferroptosis in cochlear hair cells through multi-target mechanisms of action, demonstrating promising otoprotective potential (31).

Chiisanoside is a lupane-type triterpenoid compound extracted from natural plants. Experimental research has revealed its dual protective mechanisms against drug-induced hearing loss (41).

First, activating endogenous anti-ferroptosis pathways. In damage induced by ototoxic drugs such as cisplatin, Chiisanoside significantly upregulates the protein expression levels of SLC7A11 and GPX4 in cochlear hair cells (41). SLC7A11 is a key component of system Xc^−^, responsible for cystine uptake to synthesize glutathione (GSH); GPX4 is the key enzyme that relies on GSH to clear lipid peroxides. By activating this core antioxidant defense axis, Chiisanoside effectively enhances cellular resistance to lipid peroxidation damage. This process may involve the activation of TGFBR2, which plays a role in cellular stress responses (41).

Second, stabilizing the cytoskeletal structure. Beyond targeting the ferroptosis pathway, Chiisanoside also directly protects the mechanosensitive structures of hair cells by modulating actin cytoskeletal dynamics. It maintains stable actin assembly by upregulating the expression of Dock1 and PIP5K1A while inhibiting the activity of Gelsolin, collectively preventing the cytoskeletal depolymerization induced by ototoxic drugs. A stable cytoskeleton is crucial for maintaining the morphology of hair cells and their auditory transduction function (41).

This dual-mechanism action enables Chiisanoside to demonstrate more comprehensive protective effects than single-target agents in experimental models, thereby providing a novel perspective for the development of multi-target therapeutic strategies against drug-induced deafness.

Luteolin is a flavonoid compound widely present in vegetables and fruits and is an FDA-approved drug for therapeutic use. Its anti-ferroptotic effects primarily involve the regulation of iron homeostasis and the inhibition of oxidative damage.

First, Luteolin significantly reduces the expression level of intracellular transferrin, thereby decreasing cellular uptake of iron ions. This directly leads to a reduction in the concentration of intracellular free Fe^2+^ (31). Since Fe^2+^ is essential for catalyzing the Fenton reaction in lipid peroxidation, the decrease in its concentration suppresses the initiation of the lipid peroxidation chain reaction at its source (73).

Second, Luteolin possesses potent free radical scavenging ability, directly neutralizing ROS and existing lipid peroxides. Furthermore, studies indicate that flavonoids such as luteolin can intervene in the Nrf2/GPX4 signaling pathway (70, 74). By regulating this pathway, it further influences the expression of a series of downstream genes related to antioxidant stress and ferroptosis, thereby consolidating the cellular defense system.

Chiisanoside and Luteolin, as naturally derived compounds capable of inhibiting ferroptosis, provide promising candidate strategies for preventing and treating drug-induced deafness through their respective, unique, multi-faceted mechanisms. The distinctive feature of Chiisanoside lies in its dual action of simultaneously activating the endogenous antioxidant pathway and stabilizing the cytoskeleton. In contrast, Luteolin primarily focuses on modulating iron homeostasis and exerting multi-pathway antioxidant effects. Future research holds promise for combining drugs with different mechanisms of action or integrating them with nanoparticle-based drug delivery systems to enhance inner ear targeting. This approach could potentially optimize hearing protection outcomes for patients (see Table 3).

**Table 3:** Dosing regimens of ferroptosis-targeting compounds in hearing protection studies.

Compound	Target/mechanism	Model	Dosage/Regimen	Route	Duration	Ref.
Deferoxamine	Iron chelator	Cisplatin-induced ototoxicity	200 mg/kg	Intraperitoneal	7 days	(11, 67)
Ferrostatin-1	Radical-trapping antioxidant	Noise-induced hearing loss	5 mg/kg	Intraperitoneal	3 days (pre-exposure)	(59)
Liproxstatin-1	Radical-trapping antioxidant	Neomycin-induced ototoxicity	1–10 μM	*In vitro*	24 h	(47)
4-Octyl itaconate	GPX4 activator/Nrf2 agonist	Cisplatin-induced ototoxicity	25 mg/kg	Intraperitoneal	3 consecutive days	(70)
Chiisanoside	SLC7A11/GPX4 upregulator	Cisplatin-induced ototoxicity	10 mg/kg	Intraperitoneal	7 consecutive days	(41)
Luteolin	Iron homeostasis regulator	Cisplatin-induced ototoxicity	1 mg/kg (prophylactic)	Intraperitoneal	Before each cisplatin cycle	(31)

For compounds that have been studied primarily *in vitro* (e.g., Liproxstatin-1), the effective concentration and exposure time are provided. In vivo dosing for Liproxstatin-1 in hearing loss models remains to be established.

### 4.3 Novel nanotechnology and delivery systems

The development of novel nanotechnology and delivery systems provides a breakthrough solution for overcoming the blood-labyrinth barrier (BLB) and achieving efficient, targeted drug delivery to the inner ear. Among these, single-atom nanozymes and conductive sustained-release hydrogels stand out as two representative platforms with significant application potential.

Single-atom nanozymes anchor metal active centers onto a support in the form of isolated atoms, achieving maximum atomic utilization and uniform catalytic active sites. For instance, a bimetallic copper-manganese single-atom nanozyme (Cu-Mn NE) has been designed to mimic the activities of superoxide dismutase (SOD) and catalase (CAT). Within its structure, the incorporation of manganese optimizes the electronic structure of copper, facilitating electron transfer. This not only enhances its adsorption capacity for ROS but also significantly lowers the energy barrier for catalytic reactions. This synergistic effect enables this nanozyme to scavenge ROS with far greater efficiency than its monometallic counterparts (75).

In the context of neomycin-induced hair cell damage, such nanozymes efficiently convert superoxide anions (O₂•^−^) into hydrogen peroxide (H₂O₂) and subsequently decompose H₂O₂ into harmless water and oxygen (75). This cascading catalytic reaction effectively interrupts the ROS-induced chain reaction of lipid peroxidation, thereby mechanistically inhibiting the ferroptosis process and consequently providing robust protection for hair cells (75).

Conductive hydrogels represent another distinctive strategy, combining the capability for controlled drug release with responsiveness to electrical stimulation. This makes them particularly well-suited as a delivery platform for “drug cocktails” intended for localized, sustained-release therapy within the inner ear.

Taking poly(2-isopropenyl-2-oxazoline) (PiPOx) hydrogel as an example, the oxazoline rings within its network structure can establish diverse non-covalent interactions (such as ionic, hydrogen bonding, and hydrophobic interactions) with various drug molecules. This enables efficient loading of drugs with different properties and precise control over their release kinetics. Furthermore, its pH-responsive properties allow for the intelligent release of drugs in specific microenvironments, such as the acidic conditions commonly found in inflamed tissues. This characteristic enables it to co-load ferroptosis inhibitors, neurotrophic factors, and anti-inflammatory drugs, synergistically protecting auditory cells via multiple mechanisms (76, 77).

On the other hand, conductive hydrogels prepared from a composite of polyvinyl alcohol (PVA), Laponite (LAP) clay, and the conductive polymer PEDOT: PSS exhibit excellent conductivity (up to 17.9 S/m) and mechanical properties. Upon application of an external electric field (EF), this hydrogel enables on-demand, rapid drug release (e.g., releasing up to 86% of the model drug diclofenac sodium within 3 h). This provides the possibility of precisely controlling intracochlear drug concentrations via external stimuli when needed (78).

Single-atom nanozymes directly scavenge ROS by efficiently mimicking endogenous antioxidant enzyme mechanisms, whereas conductive sustained-release hydrogels provide a platform for achieving long-lasting, synergistic cochlear protection through their intelligently controllable drug release and electrophysiological modulation. Although the clinical translation of these technologies still faces intricate challenges, they undoubtedly open up promising new directions for the future development of highly effective and precise strategies for the prevention and treatment of deafness.

### 4.4 Exploration of multi-target modulation strategy: a case study of traditional Chinese medicine treatment

Ferroptosis is regulated by a complex network, suggesting that multi-target modulation strategies may offer advantages over single-node interventions (79, 80). Traditional Chinese Medicine (TCM), with its holistic philosophy and multi-component synergy, has long been used in managing hearing loss and may align with the concept of ferroptosis network modulation (81).

Certain herbal compounds and acupuncture have demonstrated broad pharmacological effects relevant to inner ear protection, including anti-inflammatory, antioxidant, and microcirculatory actions (82). These effects could potentially converge on ferroptosis-related pathways such as the System Xc^−^-GSH-GPX4 axis or iron homeostasis (83–85). For instance, ligustilide has been suggested to modulate ferroptosis-related proteins in the auditory cortex, and acupuncture at points like Yifeng and Tinghui may reduce oxidative stress and improve microcirculation, thereby creating an environment less permissive for ferroptosis (86).

It is essential to clearly recognize that the direct mechanistic links between TCM treatments and ferroptosis currently remain largely hypothetical and in exploratory stages, far from achieving clear molecular-level elucidation. However, this intervention philosophy based on a “multi-target-complex network” approach offers a novel research direction distinct from single highly selective inhibitors (87). Future studies employing modern pharmacological and molecular approaches are needed to clarify the specific targets and pathways of TCM components in SNHL models, which could ultimately integrate traditional insights with contemporary ferroptosis-based therapeutic strategies.

## 5 Core scientific controversies and translational bottlenecks

Although the role of ferroptosis in SNHL has been widely substantiated, considerable controversy persists regarding its specific molecular mechanistic network.

### 5.1 Mechanistic controversies: causal primacy vs. secondary activation

A central unresolved question is whether ferroptosis acts as a primary driver of cochlear cell death or is merely secondarily activated following other forms of injury. Current research often relies on the use of specific inhibitors (e.g., Ferrostatin-1) to infer the contribution of a particular pathway. However, crosstalk and compensatory mechanisms exist among cell death pathways. Inhibiting one pathway may lead cells to switch to alternative modes of death, thereby potentially causing misinterpretation of the role of a single pathway. Dissecting these intertwined pathways requires moving beyond inhibitor-based correlative studies toward genetic models with cell-type-specific, inducible knockout of core ferroptosis regulators (e.g., GPX4, ACSL4) while dynamically monitoring multiple cell death modalities. Such approaches would clarify whether ferroptosis operates upstream as an initiating event or downstream as an amplifying loop.

Another core controversy lies in whether the initial triggering signals of ferroptosis induced by different etiologies (such as cisplatin-induced ototoxicity, noise exposure, and aging) share a common pathway or possess distinct activation mechanisms (88, 89). Understanding these similarities and differences is crucial for developing broad-spectrum or precisely personalized therapeutic strategies (90). As systematically compared in Section 3.4 and Table 1, different etiologies have their own emphases in the upstream triggering events (e.g., the source of iron dysregulation, the initial stress signals). This “convergent downstream, divergent upstream” architecture has important therapeutic implications. Broad-spectrum strategies targeting shared execution machinery (e.g., lipid peroxide scavenging) may be effective across etiologies, whereas etiology-specific interventions (e.g., preventing cisplatin-induced NCOA4 activation) may offer precision benefits but require diagnostic stratification. The field currently lacks comparative studies directly interrogating whether these pathways are truly convergent or whether etiology-specific nuances demand tailored approaches.

Furthermore, two critical questions remain unresolved in this field. On one hand, what are the specific patterns of “crosstalk” between ferroptosis and other cell death pathways? (91) For instance, does the activation of inflammasomes associated with pyroptosis provide substrates for lipid peroxidation in ferroptosis, or do membrane rupture signals in necroptosis accelerate the propagation of ferroptosis? Uncovering the specific molecular bridges underlying these intertwined reactions is crucial for understanding the complex mechanisms of SNHL damage. On the other hand, the greatest obstacle in transitioning from basic research to clinical application is the lack of large animal models and direct human evidence (92). Current conclusions are largely based on rodent models such as mice and rats, which inherently differ from humans in lifespan, cochlear fine structure, and metabolic homeostasis. Due to the scarcity of direct data from non-human primates or human inner ear tissues (e.g., temporal bone specimens), it remains unclear to what extent ferroptosis contributes to human SNHL and whether the core regulatory networks are fully conserved across species. This evidence gap represents a fundamental void that must be addressed in future translational research.

### 5.2 Translational bottlenecks: beyond preclinical promise

Translating ferroptosis-targeting strategies to clinical application faces multi-layered challenges that extend beyond basic mechanistic questions.

Firstly, there is the dilemma of missing biomarkers and an unclear therapeutic time window (93). Is the optimal timing for intervention preventive administration or salvage treatment after injury has occurred? This is difficult to determine in clinical practice, largely because we lack non-invasive biomarkers that can be used for *in vivo*, dynamic monitoring of cochlear ferroptosis activity (94). Additionally, the long-term safety and tolerability of the vast majority of ferroptosis inhibitors in humans have not been systematically evaluated, and their potential long-term effects remain unknown (95).

Secondly, there is the systemic obstacle of targeted delivery to the inner ear. The blood-labyrinth barrier (BLB) protects the cochlear microenvironment but excludes most systemically administered ferroptosis inhibitors (iron chelators, lipophilic antioxidants) (96). Achieving therapeutic concentrations in hair cells and spiral ganglion neurons while avoiding systemic toxicity (e.g., systemic iron deficiency from deferoxamine) requires innovative delivery strategies. Intratympanic injection bypasses the BLB but suffers from rapid drug clearance via Eustachian tube drainage and variable round window permeability. Nanocarrier systems (liposomes, nanoparticles, hydrogels) offer potential for sustained release and cellular targeting, but their long-term biosafety, manufacturing scalability, and regulatory approval pathways remain unresolved (60, 97–100). The field must rigorously evaluate whether these advanced delivery platforms can achieve therapeutically relevant intracochlear pharmacokinetics with acceptable safety profiles.

Thirdly, the predictive validity of preclinical models for human SNHL is uncertain. Most efficacy data derive from rodent acute injury models, which compress pathophysiological timecourses (days to weeks) that unfold over years or decades in human disease—particularly age-related hearing loss. This “timescale compression” may overestimate the efficacy of acute interventions for chronic conditions. Furthermore, rodents differ from humans in cochlear size, frequency range, metabolic rate, and lifespan, raising concerns about cross-species translatability. The scarcity of large animal (non-human primate) studies and the near-absence of human inner ear tissue validation create a fundamental evidence gap that undermines confidence in clinical predictions (101–103). Table 2 systematically compares model systems and their limitations, highlighting the urgent need for human-relevant platforms such as cochlear organoids and induced pluripotent stem cell-derived hair cells.

Fourthly, the translational pathway lacks clear regulatory and clinical development frameworks. Ferroptosis inhibitors for SNHL will likely require orphan drug designation and rely on well-defined clinical endpoints (e.g., pure-tone audiometry changes) and validated biomarkers to design pivotal trials. Defining a de-risked translational roadmap—from lead optimization through IND-enabling studies to early proof-of-concept trials—is an urgent priority. This includes addressing long-term safety, potential off-target effects (e.g., interference with physiological iron homeostasis), and formulation stability for inner ear administration.

Fifthly, potential interactions with current treatments must be carefully considered. Systemic ferroptosis inhibition could theoretically reduce the anti-tumor efficacy of cisplatin, which partly relies on ferroptotic stress in cancer cells (34, 104). This therapeutic dilemma underscores the need for inner ear-targeted delivery (e.g., intratympanic injection, nanocarriers) or sequential dosing (initiating inhibition after chemotherapy) to preserve otoprotection without compromising anti-tumor effects. Co-administration with aminoglycosides may offer protective benefits but requires evaluation of potential pharmacodynamic interference with antibacterial activity. Corticosteroids, commonly used for sudden hearing loss, have been shown to modulate ferroptosis susceptibility—for instance, dexamethasone sensitizes cells to ferroptosis via glucocorticoid receptor-induced glutathione depletion (105). This suggests potential synergy with ferroptosis inhibitors, a combination strategy warranting further investigation (52).

Sixthly, safety considerations for long-term systemic inhibition warrant attention. Beyond its pathological role, ferroptosis serves important physiological functions in host defense and tumor suppression, including the elimination of infected or oncogenic cells (106). Long-term systemic administration of ferroptosis inhibitors could theoretically increase infection susceptibility and cancer risk. Therefore, clinical development should prioritize local, inner ear-targeted delivery strategies that achieve therapeutic concentrations in the cochlea while minimizing systemic exposure, thereby preserving the beneficial roles of ferroptosis in systemic physiology.

In summary, while the preclinical rationale for targeting ferroptosis in SNHL is compelling, critical appraisal reveals substantial gaps in causal evidence, biomarker availability, delivery technology, and model validity. Closing these gaps will require not only mechanistic refinement but also strategic investment in translational tools and interdisciplinary collaboration.

## 6 Conclusions and future perspectives

In summary, this review has discussed how ferroptosis, an iron-dependent, lipid peroxidation-driven form of regulated cell death, has been established as a crucial pathogenic pathway in various forms of acquired sensorineural hearing loss (SNHL). By systematically synthesizing the body of evidence linking molecular mechanisms to therapeutic interventions, this review fully demonstrates that targeting ferroptosis represents a highly promising novel therapeutic strategy.

At the mechanistic level, ferroptosis is intricately linked to SNHL through a sophisticated yet vulnerable regulatory network. Its core lies in the disruption of intracellular redox homeostasis, specifically manifested by the collapse of three major defense systems: the inactivation of the GPX4/GSH core system, the inadequate compensation of the FSP1/CoQ10 backup system, and the ACSL4/LPCAT3-mediated reprogramming of lipid metabolism. Across various etiologies of acquired SNHL—whether it be the direct toxicity of cisplatin and neomycin, the energy metabolism crisis induced by noise exposure, or the cumulative oxidative damage from aging—these stressors ultimately converge at a common endpoint: the uncontrolled accumulation of lethal lipid peroxides in cochlear hair cells and spiral ganglion neurons. Notably, the elucidation of the FOXO1-NCOA4 axis-regulated ferritinophagy mechanism tightly links iron metabolism dysregulation with the failure of antioxidant defense, explaining why cochlear cells are so vulnerable to specific injuries. This mechanistic convergence provides a theoretical foundation for developing broad-spectrum protective drugs.

Correspondingly, the intervention strategies exhibit characteristics of being multi-target and multi-level. From Ferrostatin-1 and Liproxstatin-1, which directly neutralize lipid radicals, to the iron chelator deferoxamine, which reduces catalytic iron sources; from GPX4 activators that enhance endogenous antioxidant capacity, to natural compounds like Chiisanoside and Luteolin that simultaneously stabilize the cytoskeleton and regulate the ferroptosis pathway—a series of preclinical studies have collectively demonstrated that intervening at key nodes of ferroptosis can effectively mitigate hearing loss. Even more groundbreaking is the emergence of novel delivery systems such as single-atom nanozymes and conductive sustained-release hydrogels, which offer hope for overcoming the historic challenge of the blood-labyrinth barrier. This represents a leap from addressing “whether intervention is possible” to “how to intervene efficiently.”

However, we must soberly recognize that this field currently remains predominantly in the preclinical research stage, and advancing toward clinical translation still faces a series of formidable challenges. These challenges encompass not only unresolved mechanistic interactions but also the core application-oriented bottlenecks systematically outlined earlier: the lack of non-invasive biomarkers, issues with the efficiency and safety of inner ear targeted delivery, the predictive limitations of preclinical models, and the absence of a clear clinical translation pathway. Overcoming these bottlenecks is the key to realizing the proposed “translational prospects.”

Looking ahead, research on targeting ferroptosis for the prevention and treatment of SNHL is poised to achieve significant breakthroughs in the coming decade. These advancements will rely on deeper interdisciplinary integration. Gene editing technologies and cell-type-specific animal models will help us precisely dissect the dynamic process of ferroptosis in specific cochlear cell populations. Single-cell sequencing and spatial transcriptomics hold the promise of unveiling unique ferroptotic signatures across different SNHL subtypes, providing a basis for patient stratification. AI-based drug design will accelerate the development of novel, highly effective, and low-toxicity ferroptosis inhibitors. Meanwhile, progress in nanotechnology and materials science will continue to optimize the delivery efficiency and controlled-release performance of drugs to the inner ear.

In summary, we can foresee the advent of a new era of precise and personalized prevention and treatment of hearing loss. By integrating multi-omics data, clinicians will be able to identify a patient’s “ferroptosis risk signature,” thereby formulating targeted prevention or treatment plans. As mechanistic studies continue to clarify and new technologies persistently emerge, the strategy of targeting ferroptosis holds the promise of evolving from current experimental research into a mature clinical therapeutic approach. Ultimately, through a close loop of basic discoveries and translational medicine, the strategy of targeting ferroptosis holds high potential to evolve from current experimental research into a mature clinical therapeutic approach, offering new hope to hundreds of millions of patients with SNHL.
